# Neonatal Bowel Obstruction by Pseudolymphoma: A Case Report

**DOI:** 10.7759/cureus.13746

**Published:** 2021-03-06

**Authors:** Apostolia Galani, Athanasios Zikopoulos, Eirini Mastora, Konstantinos Zikopoulos

**Affiliations:** 1 Obstetrics and Gynaecology, University Hospital of Ioannina, Ioannina, GRC; 2 Obstetrics and Gynaecology, Royal Cornwall Hospital, Cornwall, GBR

**Keywords:** fetal interstitial obstruction, pseudolymphoma

## Abstract

Interstitial obstruction in newborn infants can be caused by several factors such as malrotation, meconium plug syndrome, meconium ileus, Hirschsprung's disease, atresia and stenosis. Neonates who have been diagnosed with an interstitial obstruction are in need of immediate treatment; otherwise, they can deteriorate rapidly. Surgery remains the mainstay of treatment in most cases. Pediatric gastrointestinal tumours are very rare, especially in newborn infants. Their management is usually different as compared to adults.

We present the case of a newborn infant who was born with interstitial obstruction. At the 31 weeks scan, a significant dilation of the small bowel was observed and the diagnosis of interstitial obstruction was made. When born, the newborn was transferred to a specialised unit and underwent a laparotomy. The findings were consistent with a tumour causing the obstruction; the histology reported this tumour as benign lymphoid hyperplasia.

Pseudolymphoma is a very rare cause of fetal interstitial obstruction, and it should be considered in the differential diagnosis.

## Introduction

The gastrointestinal tract is divided into two segments: the small intestine and the large intestine, known as the colon. The small intestine is made up of three parts: the duodenum (which is connected to the stomach), the jejunum and the ileum.

An interstitial obstruction in a newborn infant can be caused due to several conditions such as atresia and stenosis, annular pancreas, volvulus (twisting of the bowel on itself), duplication cyst, meconium ileus, meconium plug syndrome, Hirschprung’s disease, neoplasia, trauma and some other rare causes such as necrotising enterocolitis [1]. Neonates with unrecognised intestinal obstruction deteriorate rapidly and in these cases, mortality and morbidity can be high. Therefore, early diagnosis is crucial.

A fetal bowel obstruction is usually discovered in two ways. A routine scan during gestation (usually after 20 weeks of gestation) may show that a certain segment of the bowel is abnormal, which could be either dilated or larger than normal. While in the uterus, the fetus swallows amniotic fluid. Therefore, a narrowing of the gastrointestinal system could slow or even stop the flow of the amniotic fluid, causing swelling of the bowel, which will make the bowel appear large in the scan. In this case, the normal movements the bowel makes become exaggerated as the bowel tries to propel fluid through the blockage. These movements, also called peristalsis, can be seen over several seconds with ultrasound. A bowel obstruction could also be discovered when polyhydramnios is present in a scan. Because of the obstruction, the normal flow of the amniotic fluid will be stopped. Subsequently, the amniotic fluid stays outside the fetus and inside the uterus. The uterus can be enlarged due to the high amount of amniotic fluid. If the fetus is diagnosed with an interstitial obstruction, a thorough evaluation is significant. This evaluation may include an ultrasound, fetal echocardiogram and amniocentesis.

The management of an intestinal obstruction will almost always be surgical. With the advent of neonatal intensive care and multidisciplinary care, morbidity and mortality rates are low. Tumours of the pediatric or neonatal gastrointestinal tract are extremely rare. Their infrequent presentation at treatment centres has not allowed for the development of standardized treatment protocols and prospective review [2].

## Case presentation

A 32-year-old Caucasian pregnant woman (G2, P1) presented at the antenatal department due to reduced fetal movements the day before. She was 31 weeks and four days pregnant after spontaneous conception. This was her second pregnancy. During this pregnancy, she had a 12 weeks screening and a 20 weeks scan. Both of these screenings were reassuring. Her first pregnancy was three years ago with normal delivery. The first baby was a girl who weighed 3210 grams.

**Figure 1:**
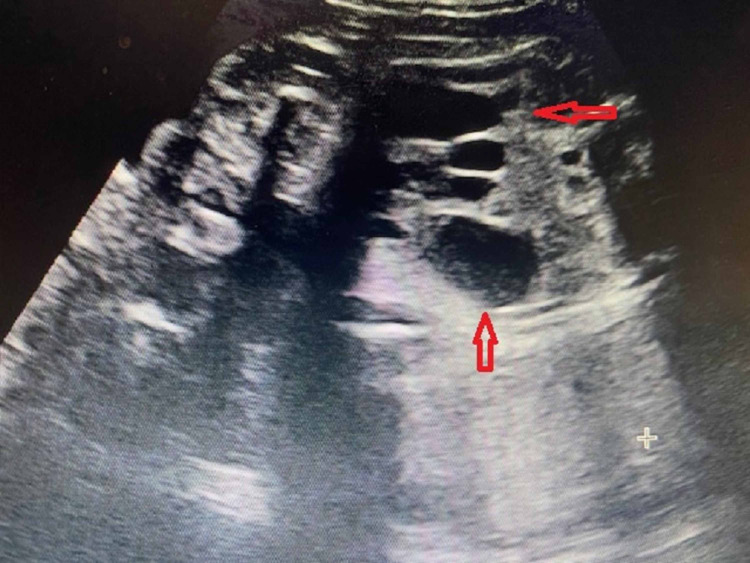
The dilated bowel loop that was observed during the scan

Initially, she had a cardiotocography (CTG), which was reassuring. The baseline was around 130 bpm with accelerations while no contractions were recorded. Afterwards, she had a scan, which was done by a consultant specialised in fetal medicine. During this scan, significant small bowel dilation was observed and the diagnosis of interstitial obstruction was made (Figure 1). The amniotic fluid index was normal (22 cm) and fetoplacental dopplers were reassuring. During the scan, the peristalsis of the fetal bowel was observed.

The patient was admitted to the antenatal unit for daily observation and assessment. On Day 2 of hospitalisation, the patient had an amniocentesis to detect any chromosome abnormalities, genetic disorders etc. The results of the amniocentesis came back as reassuring. During this time and after liaising with the neonatal unit, an obstetric scan was repeated twice weekly to monitor any bowel changes. During these scans, the bowel enlargement was stable, the bowel was not hyperechoic and peristalsis was present. Therefore, it was decided to wait until 37 weeks for labour induction unless there was a significant deterioration in either the scan findings or the CTG.

**Figure 2:**
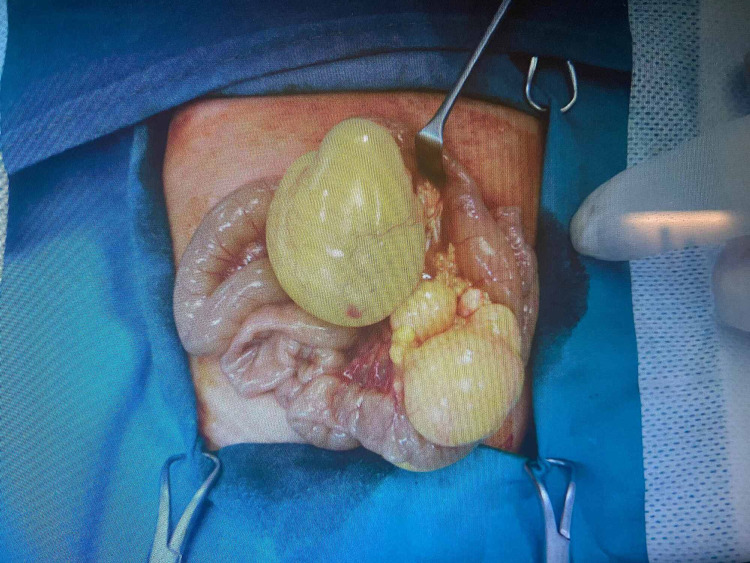
The tumour that caused the obstruction

The patient delivered at 36 weeks and two days after spontaneous rupture of membranes. A male infant who weighed 2620 grams was delivered. The newborn was transferred immediately to the neonatal intensive care unit where a group of specialised doctors assessed him. After several examinations, such as X-rays and MRI scans, the diagnosis of interstitial obstruction was established and the newborn infant was transferred to another unit with specialised surgeons after having a nasogastric tube and being stable. On Day 2 post-delivery, the newborn underwent a laparotomy. The findings of the laparotomy were as follows: a large tumour that blocked the jejunum was identified (Figure 2). A part of the bowel was necrotic. The tumour was excised and a part of the jejunum was removed. The jejunum was connected at the same surgery. The tissues were sent for histology.

The newborn boy was hospitalised for a total of 28 days. The results of the histology showed lymphoid hyperplasia, which was benign, and its possible origin was regional lymph nodes. After a multidisciplinary meeting, it was decided that no further treatment was necessary, and the male infant was to attend a follow-up appointment in three months.

## Discussion

Interstitial obstruction in a fetus is a challenging diagnosis, and it needs a direct assessment by neonatologists after the delivery [1-2].

A high interstitial obstruction may be attributable to oesophagal atresia (with or without fistula), duodenal atresia, malrotation (with or without midgut volvulus) or jejunal atresia. Low Interstitial obstruction is most commonly attributable to ileal atresia, meconium ileus, Hirschsprung’s disease, small left colon syndrome, colonic atresia or imperforate anus [3]. However, a neonatal interstitial obstruction due to a tumour is very rare even more so due to pseudolymphoma.

Benign lymphoma, known as pseudolymphoma or benign lymphoid hyperplasia, is a condition that has symptoms similar to malignant lymphomas. Benign tumours are lumps or growths that may appear in several parts of the body. In the case of benign lymphoma, these sites are usually the skin, lungs, gastrointestinal tract, soft tissues and many other sites. The reasons for the development of a benign lymphoma are not completely understood but it is associated with genetics, radiation exposure, inflammation or infections. About one in every 2,000 people worldwide suffer from benign lymphomas, which are rarely life-threatening. Pseudo lymphoma in children is extremely rare, especially in neonates, since this is one of the few cases that have been described so far. In most cases, benign lymphoma is not severe. However, it may cause problems when the swollen lymph nodes start to press into surrounding tissues, or in even more severe cases, it could evolve into a malignant lymphoma [4].

## Conclusions

Although fetal interstitial obstruction is a relatively common diagnosis that most fetal medicine doctors will have to deal with, a tumour as a causative factor is not very common. Especially, pseudolymphoma tumours are extremely rare. Therefore, it should be included in the differential diagnosis, and it should be discussed with the neonatal team so that better treatment is planned for the newborn infant.
